# Nutritional Improvement of Fresh Cheese with Microalga *Chlorella vulgaris*: Impact on Composition, Structure and Sensory Acceptance

**DOI:** 10.17113/ftb.61.02.23.7851

**Published:** 2023-06

**Authors:** Rita Lousada Falcão, Valentina Pinheiro, Cátia Ribeiro, Isabel Sousa, Anabela Raymundo, Maria Cristiana Nunes

**Affiliations:** 1 LEAF - Linking Landscape, Environment, Agriculture and Food Research Center, Associated Laboratory TERRA, School of Agriculture, University of Lisbon, Tapada da Ajuda, 1349-017 Lisboa, Portugal; 2 Queijos Santiago, Montemuro Apartado 51, 2669-909 Malveira, Portugal

**Keywords:** microalgae *Chlorella vulgaris*, fresh cheese, nutritional properties, texture profile analysis

## Abstract

**Research background:**

The production of foods fortified with bioactive ingredients has been recognized by food companies as a way to position their products in health food markets. The fortification of cheese represents a major challenge, due to the chemical and structural complexity of the cheese matrix, as well as the complexity of the biochemical reactions occurring during the fermentation and maturation processes. Microalgae are nutritious and sustainable food sources with important bioactive compounds such as proteins, polyunsaturated fatty acids, polysaccharides, chlorophylls, carotenoids, vitamins and minerals.

**Experimental approach:**

This work aims to study the impact of the 2 and 4 % microalga *Chlorella vulgaris* addition on the nutritional composition, bioactivity, structure and sensory profile of quark and cream cheese, both probiotic fermented products. Texture profile analysis and fundamental rheology measurements (oscillatory and stationary) were performed to evaluate the impact of *C. vulgaris* on the mechanical properties of the fresh cheese. The nutritional composition was evaluated using standard methods and bioactivity through the determination of total phenolic compounds and antioxidant capacity.^1^

**Results and conclusions:**

*C. vulgaris* had an impact on the firmness of both cheeses. In general, the cheese with added *C. vulgaris* had a better nutritional profile, with an increase in protein content, content of Mg, P, S, Cu, Zn, Fe and Mn, and better bioactivity with an increase in the antioxidant activity. Sensory testing results were promising, especially for cream cheese.

**Novelty and scientific contribution:**

The enrichment of traditional foods such as fresh cheese with microalgae represents an interesting strategy to develop hybrid products (with protein from animal and vegetable sources), obtain innovative and more sustainable products, and improve their nutritional profile in terms of protein and mineral content and bioactivity.

## INTRODUCTION

Microalgae are a group of microscopic organisms that can be found in a wide variety of aquatic environments. Most microalgae are classified as eukaryotic microorganisms, but some prokaryotes, like cyanobacteria, can also be characterized as such. Microalgal biomass can have several applications, from improving human and animal nutrition through additions to food and feed to wastewater treatment and bioenergy (*1*, *2*). Some microalgae (*Aphanizomenon flos-aquae*, *Arthrospira platensis*, *Chlorella luteoviridis*, *Chlorella pyrenoidosa* and *Chlorella vulgaris*) were already regularly consumed in the European Union (EU) before 1997. *Chlorella vulgaris* is not considered as a novel food. Therefore, there is no need to go through the (EU) Regulation 2015/2283 of the European Parliament and of the Council of 25 November 2015 on novel foods (*1*, *2*) and it can be used as a food ingredient.

*Chlorella vulgaris* demonstrates several characteristics that make it a better candidate for the use in food than other microalgae. It mainly multiplies under autotrophic conditions but can also be produced under heterotrophic or mixotrophic conditions (*3*). Its composition is very dependent on growing conditions, namely lighting and growth medium composition. For example, Gouveia *et al*. (*4*) reported the following composition of autotrophic *C. vulgaris*: protein 51-58 %, polysaccharides 12-17 % and lipids 14-22 %. In addition to the macronutrients, it has important bioactive compounds such as essential amino acids, polyunsaturated fatty acids (PUFAs), polysaccharides, chlorophylls, carotenoids, vitamins and minerals (*5*, *6*). Due to its chemical composition, numerous benefits of this microalga for human health have been reported, such as antitumour properties of some of its glycoproteins, peptides and nucleotides, promoting factors against gastric ulcers, wounds and constipation (*4*). It also contains a mixture of fatty acids (chlorellin), with inhibitory activity against both Gram-positive and Gram-negative bacteria, so *C. vulgaris* can be used as a natural antibiotic (*7*).

In recent years, researchers have been interested in micro- and macroalgal biomass due to its remarkably rich composition. Studies have been conducted on the incorporation of algae in dairy products (*8*) such as milk (*9*, *10*), yoghurt (*11*) and other fermented kinds of milk (*12*), and different types of cheese, processed cheese analogue (*13*), spreadable cheese (*14*), ricotta cheese (*15*) and semi-hard cheese (*16*). In a recent paper, *Arthorspira platensis* (spirulina) was incorporated into feta-type cheese to study its effect on microflora and the physicochemical properties of the cheese (*17*).

The demands and needs of consumers are changing over time. For these needs to be met, it is necessary to study consumer trends, so that companies can innovate and adapt their products accordingly. As stated by Sloan (*18*) the increase in demand for functional foods that contribute to a healthy diet and help prevent diseases has been noticeable in recent times, with increased consumer awareness of the impact of food on health and well-being. Thus, the incorporation of bioactive ingredients, such as microalgae, which are rich in antioxidants and several bioactive compounds (pigments, vitamins, minerals, PUFAs) already familiar to the consumer, into foods has been the focus of several different companies in the food industry. By incorporating these ingredients, companies can create functional foods that respond to consumer desires.

Besides the health benefits, consumers often seek new flavours in dairy products. During the COVID-19 pandemic, many consumers continued their love of exploring the worlds through their taste buds, seeking new tastes paired with approachable and familiar formats and flavours, for example, key lime-flavoured yoghurt or barbecue flavoured unripened firm cheese (*19*). The addition of microalgae and their characteristic flavour to a traditional product like cheese measures up to this trend.

Smooth *C. vulgaris* is a variant that can be obtained through heterotrophic production. This production consists of the exclusive use of organic compounds as a carbon source by the microalga for its development and biomass production. The great advantage of this type of production over the autotrophic production is that it is faster and takes less space to produce the same amount of biomass (*20*). Some studies have used agricultural by-products and food wastes as low-cost substrates for the heterotrophic species, showing the low environmental impact of this cultivation (*21*, *22*). In this work, smooth *C. vulgaris* was chosen for incorporation into the fresh cheese due to its light green colour and milder sensory characteristics to overcome barriers to consumption and facilitate its use as a food ingredient. This work is part of the project AlgaeGreenCheese, developed by Queijos Santiago company, a Portuguese market leader. It aims to study the impact of the incorporation of smooth *Chlorella vulgaris* (from *w*=2 to 4 %) on the structure, nutritional composition, bioactivity and sensory profile of quark and cream cheese.

## MATERIALS AND METHODS

### Cheese manufacture

The fresh cheese types used in the present work were produced at Queijos Santiago’s Innovation Laboratory facilities (Mafra, Portugal), using a flow chart and raw materials identical to those of industrial production. Spray-dried smooth *Chlorella vulgaris* was provided by Allmicroalgae S.A. (Pataias, Portugal) and was obtained through heterotrophic production.

Quark cheese was manufactured by adding 2 and 4 % (*m*/*V*) of microalga to pasteurized cow’s milk at 80 °C. It was mixed, homogenized and left to cool until the desired temperature of 37 °C to add the lactic acid starter cultures (*Lactococcus*). Afterwards, the cultures were added, and the vat was placed in the incubator at 37 °C for about 16 h to coagulate/ferment. After the acid coagulation, with lactic bacteria, the curd was cut at approx. 1 cm and drained using draining cloths. The quark mass was once again mixed and homogenized using a stick mixer 400 W (Braun, Kronberg im Taunus, Germany) at 13 900 rpm and finally, it was placed in cuvettes and stored at refrigeration temperature. The control sample was quark cheese made from pasteurized cow's milk and starter cultures, without the addition of microalga.

The production of the cream cheese started with the addition of lactic acid starter cultures to the pasteurized cow’s milk, which is responsible for the acid coagulation. The milk was left to ferment for 14 h at 37 °C. Afterwards, the fermented curd was cut at approx. 1 cm, drained, homogenized and heated. Pasteurized cream, dairy proteins, acidity regulators, anti-caking agents, stabilizers/hydrocolloids and salt were added, according to Queijos Santiago´s formulation, and the mass was stirred and mixed at 95 °C. The amount of 2 and 4 % (*m*/*V*) microalga was added to the cheese mass at 90 °C and homogenized using a hand blender 300 W (Electric Co, Lisbon, Portugal) at medium speed for 1.5 min. The blend was poured into plastic containers, sealed and stored at refrigeration temperature (0–5 °C). The control cream cheese was prepared using the same ingredients and following the described procedure without the addition of microalgae. The stabilizers are important ingredients in this formulation that impact the texture of this cheese and give it a better consistency than that of the quark.

### Nutritional composition analysis

Protein mass fraction was determined by total combustion of the sample matrix under oxygen according to the Dumas method (*23*), using a Vario EL elemental analyser (Elementar, Langeselbold, Germany), and weighing the mass difference of approx. 100 mg of each sample in triplicate before and after combustion. Fresh sample was weighed directly on the foil used for analysis.

The total fat content was determined according to AACC Method 30 (*24*). In short, fresh sample was weighed directly in Erlenmeyer flasks (100 g), in triplicate. Afterwards, 7 mL of a *V*(methanol):*V*(chloroform):*V*(HCl)=10:1:1.5 (Sigma-Aldrich, Merck, Darmstadt, Germany) solution were added to the flasks, placed on a hot plate at 90 °C for 2 h and stirred with a magnet. Later, the solution was transferred to a Falcon tube, and 3 mL of *V*(hexane):*V*(chloroform)=4:1 (Sigma-Aldrich, Merck) were used to wash the Erlenmeyer flasks. The tube was vortexed for 2 min and then centrifuged for 10 min at 5478×*g* at 20 °C. After centrifugation, the supernatant was removed and transferred to a test tube, and the previous three steps were repeated. Finally, the tubes were left in the oven at 45 °C until the content was dry and then weighed. To calculate the fat mass fraction in the cheese samples, the mass of the empty test tubes was subtracted from the mass of the test tubes with dry sample.

Ash content and moisture were assessed according to the methods described by Reddy and Miravete (*25*), based on gravimetric measurements; ash content was evaluated by incineration at 550 °C in a muffle furnace (Snol, Narkunai, Lithuania) for 7 h and moisture was determined by heating at 105 °C in an oven for 3 days. For cooling, the samples were placed on an exicator for 30 min. The results were expressed in g of humidity per 100 g of sample.

Total carbohydrate mass fraction was expressed in g/100 g, and calculated by difference from the other macronutrients:


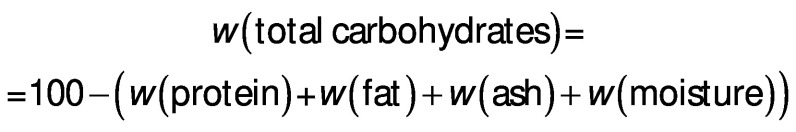
 /1/

The total energy value (*E*) was calculated based on its chemical composition and expressed in kJ/100 g (*26*):


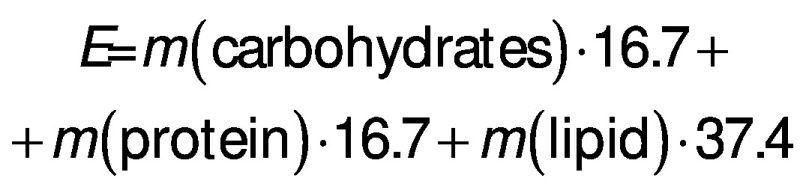
 /2/

The mineral profile was analysed using the method by Leitão *et al*. (*27*), combining absorption spectrometry and an inductively coupled plasma optical emission spectrometry (ICP-OES) Thermo Scientific iCap Series-7000 (Thermo Fisher Scientific, Waltham, MA, USA). To obtain the quantitative determination of the elements, acid digestion of the material was performed. Approximately 0.5 g of each sample was weighed directly in a tube and 12 mL of 37 % HCl and 4 mL of 65 % HNO_3_ (both Honeywell, Budapest, Hungary) were added to the tube for digestion overnight. The digested samples were placed in the extractor with the lids half open, plus two blanks, for one day, and then all were transferred to 25-mL volumetric flasks and made up to the volume with deionized water. At last, the samples were transferred to amber glass vials until they were read. The results were expressed in mg/100 g (*27*).

The pH of the samples was measured by a pH 7 Vio potentiometer (XS Instruments, Carpi, Italy) according to the method by Instituto Adolfo Lutz (*28*). It was measured at four different stages: raw milk, milk with microalgae, beginning of shelf life and the end of shelf life.

For total phenolic content (TPC) and antioxidant capacity quantification, extracts were prepared according to the procedure used by Barreira *et al*. (*29*). Briefly, 2 g of each sample were dissolved in 10 mL of ethanol (Sigma-Aldrich, Merck) and homogenized. An extraction was carried out in a water bath at 20 °C for 4 h with stirring at 150 rpm. Afterwards, the samples were centrifuged at 4025×*g* for 10 min and the supernatant was separated and stored at 4 °C. The previous steps were repeated totalling two extractions for each sample. Next, the supernatants were filtered using a syringe and placed, protected from light, on previously washed, dried and weighed balloons. The solvent was removed under vacuum using a rotary evaporator with B-491 bath (Buchi Ibérica, Barcelona, Spain) at 35 °C, and the balloons with the dry extract were weighed. Ultimately, the concentrated extract was redissolved in dimethyl sulfoxide (DMSO) (Merck, Darmstadt, Germany) to obtain the final concentration of 20 mg/mL for the cheese sample and 5 mg/mL for the smooth *C. vulgaris* sample.

TPC was determined using the Folin-Ciocalteu assay as described by Mohankumar *et al*. (*30*). In short, 150 µL of the extract were added to 140 µL of Folin-Ciocalteu reagent (Merck) and 2.4 mL of deionized water, stirred in the vortex, put away for 3 min and then 300 µL of Na_2_CO_3_ were added. The solution was stirred in the vortex for the second time and placed in the dark at room temperature for 2 h. The absorbance was read at 725 nm against a water blank using a Cary 4000 UV-Vis spectrophotometer (Agilent Technologies, Santa Clara, CA, USA).

The antioxidant activity of the sample extracts was determined using the 2,2-diphenyl-1- picrylhydrazyl (DPPH) assay as described by Brand-Williams *et al*. (*31*), and Fe(II) reducing antioxidant power (FRAP) test, according to the Ebner *et al*. (*32*) method. For the former, 100 µL of the extract were added to 3.9 mL of prepared DPPH (Merck) solution, stirred in the vortex, and placed in the dark at room temperature for 40 min. The absorbance was read at 515 nm against a methanol (Honeywell) blank. For the latter, 90 µL of the extract were added to 270 µL of distilled water and 2.7 mL of FRAP reagent (*V*(TPTZ):*V*(FeCl_2_):*V*(acetate buffer)=1:1:10), stirred in the vortex and placed in a water bath at 37 °C for 30 min, protected from light. The absorbance was read at 595 nm against a distilled water blank.

For all the assays, each sample was analysed in triplicate. The results were expressed in mg of gallic acid equivalents (GAE) per g of extract for TPC using the equation based on the calibration curve, prepared with gallic acid. For both DPPH and FRAP assays, the results were expressed on dry mass basis in mg of Trolox equivalents (TE) per g of extract using the equation based on the calibration curve prepared with Trolox.

### Cheese structure characterization

For texture analysis, a TA.XTplus Texture Analyzer (Stable Micro Systems Ltd, Godalming, UK) with a load cell of 5 kg was used. Both types of cheese were analysed at (20±1) °C in a room with temperature control, allowing the cheese samples to equilibrate for 2 h. A texture profile analysis (TPA) was performed using a cylindrical *d*=38-mm probe for the quark cheese and a 19-mm probe for the cream cheese. The probe speed was 1 mm/s and the time between bites was 5 s for both types of cheese. The probes plunged 20 and 10 mm in the quark and cream cheese, respectively. TPA test was performed at least in triplicate for each sample.

Rheological properties of quark were studied using the Thermo Haake Mars III controlled-stress rheometer (Haake MARS III, Thermo Fisher Scientific) at 5 °C, coupled with a Peltier system for temperature control. The rheometer was equipped with a serrated lower plate of *d*=35 mm (TMP35 S) and a P35 serrated upper plate with a 2 mm gap. Each measurement was carried out in triplicate. Prior to the analysis, all the quark samples were given a slight stir to homogenize and the sample was immediately placed on the plate for analysis. A waiting time of 10 min, previously determined by a time sweep test, was applied after placing the cheese samples in the measuring device and before testing for recovery and temperature stabilization. All determinations were repeated at least three times to ensure that reproducible results were obtained. All samples were analysed at the beginning and end of shelf life.

To obtain the flow curves and the viscosity parameters of the quark cheese, the variation of stress and shear rate was measured. Prior to the analysis, all the quark samples were given a slight stir to homogenize, and the sample was immediately placed on the plate for analysis. Steady-state flow measurements were carried out using a logarithmic ramp of stresses increasing in 30 min from 0.01 to 100 Pa. The flow curves were satisfactorily fitted by the Cross equation, using TRIOS software (*33*). The comparison of the flow curve parameters resulted from the adjustment of the Cross equation (*34*):


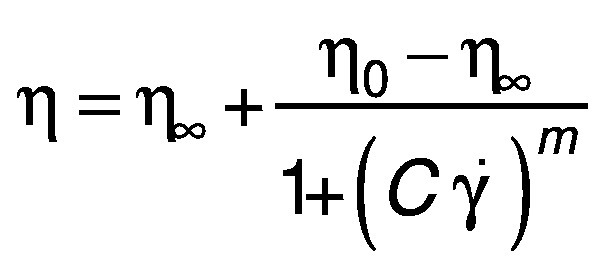
 /3/

where Cross *η*_0_ is the zero shear rate viscosity, *η_∞_* is the infinite shear rate viscosity, *m* is the Cross rate constant, *C* is the relaxation time and *γ̇ *is a shear rate.

In order to infer the microstructure and to predict the stability of the cream cheese, the linear viscoelasticity properties were studied using SAOS (small amplitude oscillatory shear) measurements. For each sample, stress sweep tests were carried out at *f*=1 Hz to determine the linear viscoelastic region. The stress value *τ*=10 Pa from the viscoelastic zone was chosen to run frequency sweep tests, ranging frequency from 0.01 to 100 Hz. The plot of viscoelastic moduli (*G’* and *G*’’) *versus* frequency was obtained, corresponding to the mechanical spectrum.

### Colour analysis

Colour was instrumentally analysed by a Chroma Meter CR-400/410 colourimeter (Konica Minolta, Tokyo, Japan), based on the CIELAB colour coordinate system (L*, a* and b*) for colour characterization to quantify the total colour differences between the control and the cheese samples with microalgae. For both types of fresh cheese (quark and cream), the Light Projection Tube CRA33B (Konica Minolta) was used. Each sample was measured in triplicate, at the beginning and the end of shelf life to study the colour stability of the cheese over time.

### Sensory evaluation

A sensory analysis was conducted on the two types of cheese - quark cheese and cream cheese - using untrained tasters of different ages. There was a total of 32 tasters for the quark cheese (22 female and 10 male) and 34 tasters for the cream cheese (23 female and 11 male). Only the cheese samples with incorporated smooth *C. vulgaris* were presented to the untrained panel.

The quark cheese samples were served in small cups along with a small amount of granola to simulate what the consumer would eat at home. The cream cheese was spread on top of a cracker.

The evaluation was performed in individual booths at the Sensory Analysis Laboratory of Instituto Superior de Agronomia, Lisbon, Portugal. The tests were carried out in a standardized sensory analysis room according to ISO 8589:2007 (*35*). The tasters expressed their level of appreciation for each attribute (colour, smell, taste, texture and overall acceptance) using a five-point hedonic scale, ranging from 1=very unpleasant to 5=very pleasant. A purchase intent test was also carried out by using a five-point scale from 1=would certainly not buy to 5=would certainly buy.

### Statistical analysis

Statistical analysis was performed using one-way analysis of variance (ANOVA) followed by Tukey’s test with p≤0.05 being considered statistically significant for the results with normal distribution. A non-parametric Kruskal-Wallis test was applied for the results with non-normal distribution. To determine if the population had a normal distribution or not, a Shapiro-Wilk test was conducted. Statistical analysis of the data was performed using OriginPro v. 8.0 (*36*).

## RESULTS AND DISCUSSION

### Nutritional composition of the cheese samples

The nutritional composition and estimated energy value of the quark and cream cheese samples is summarized in Table 1. There are no significant variations (p>0.05) regarding the results for moisture, ash and lipid mass fractions of the cheese samples with *C. vulgaris* compared to the control. However, cheese samples with incorporated *C. vulgaris* showed a significant (p<0.05) increase in protein mass fraction compared to control: 9.7 and 21.2 % in the quark cheese samples with the addition of 2 and 4 % *C. vulgaris* (Cv_2_ and Cv_4_), respectively, and 3.2 and 6.9 % in the cream cheese with Cv_2_ and Cv_4_, respectively. This increase in protein mass fraction was expected due to the high protein mass fraction of the microalgal dried biomass (32 g/100 g) although it was not the main objective of the cheese enrichment. For estimated carbohydrates, the enriched cheese samples had a similar content as the control, so it was concluded that the impact of the *C. vulgaris* was not relevant.

**Table 1:** Nutritional composition of smooth microalga *Chlorella vulgaris* (Cv), quark cheese and cream cheese with incorporated 2 and 4 % of *C. vulgaris* (Cv_2_ and CV_4_) compared to control cheese and microalgal biomass

	Microalga	Quark cheese			Cream cheese		
Smooth Cv	Control	Cv_2_	Cv_4_	Control	Cv_2_	Cv_4_
*E*/kJ	1756	242	237	260	1182	1167	1180
*w*(nutritional component)/(g/100 g)							
Carbohydrates	55.33	4.30	5.15	5.55	9.95	8.23	9.09
Protein	32.5±0.3	(5.4±0.6)^b^	(5.9±0.2)^a^	(6.5±0.2)^a^	(10.1±0.3)^b^	(11.2±0.2)^a^	(11.6±0.3)^a^
Lipids	7.6±0.3	(2.1±0.3)^a^	1.4±0.2	1.6±0.9	22.47	22.32	22.13
Moisture	4.4±0.1	87.4±1.2	86.75±0.01	85.53±0.06	56.6±0.9	57.4±0.6	56.3±0.9
Ash	0.13±0.00	0.86±0.05	0.81±0.03	0.86±0.03	3.0±0.3	2.96±0.02	3.07±0.06

Mean values of protein content for cheese samples with different letters in superscript are significantly different (p<0.05), For lipids, moisture and ash there are no significant differences (p>0.05). *E* and carbohydrates were obtained by calculation; therefore, ANOVA was not performed

Concerning nutritional claims, all quark cheese samples are ’rich in protein’, since at least 20 % of the energy value of the product comes from protein. All cream cheeses state the nutritional claim of ’source of protein’ since 12 % of the energy value of the product comes from protein according to Regulation (EU) no 1924/2006 of the European Parliament and of the Council on nutrition and health claims made on foods (*37*).

The mineral composition of *C. vulgaris* dried biomass compared to the quark and cream cheese samples is given in Table 2. Smooth *C. vulgaris* as a food ingredient states nutritional claims for K, Ca, Mg, P, Fe, Cu, Zn and Mn.

**Table 2:** Mineral content of smooth microalga *Chlorella vulgaris* (Cv), quark cheese and cream cheese with incorporated 2 and 4 % of *C. vulgaris* (Cv_2_ and Cv_4_) compared to control cheese and microalgal biomass. Mineral contents highlighted in grey possess a nutritional claim

		*w*/(mg/100 g)					
Na	K	Ca	Mg	P	S
Microalga	Cv smooth	93.2±2.1	670±16	240.3±6.6	96.6±2.7	1051±22	344±8
Quark cheese	Control	(41.6±0.5)^b^	(165.5±1.1)^b^	(220.0±1.8)^a^	(14.2±0.2)^b^	(123.0±1.3)^c^	(53.3±1.0)^c^
	Cv_2_	(44.3±0.5)^a^	(178.2±0.7)^a^	(223.6±1.7)^a^	(16.81±0.05)^a^	(140.2±0.9)^b^	(57.1±0.2)^b^
	Cv_4_	(38.1±0.4)^c^	(162.9±1.7)^b^	(217.2±1.2)^b^	(16.4±0.2)^a^	(150.4±2.5)^a^	(64.7±1.21^a^
Cream cheese	Control	(608±6)^a^	(146.6±4.6)^c^	254.6± 12.2	(9.1±0.3)^c^	(403±8)^c^	(84.5±2.0)^b^
	Cv_2_	(612±14)^a^	(159.5±4.3)^b^	255.5±2.1	(11.7±0.4)^b^	(434±10)^b^	(93.9±1.7)^a^
	Cv_4_	(695±12)^b^	(192.0±3.2)^a^	254.1±2.6	(15.0±0.4)^a^	(488±6)^a^	(96.3±1.2)^a^
DRI		-	300	120	56.25	105	-
		Fe	Cu	Zn	B	Mn	
Microalga	Cv smooth	7.4±0.3	0.40±0.02	15.2±0.4	0.00±0.00	4.4±0.1	
Quark cheese	Control	(0.3±0.2)^b^	(0.03±0.01)^b^	(0.80±0.01)^c^	(0.04±0.00)^a^	(0.01?±0.001)^c^	
	Cv_2_	(0.55±0.07)^a^	(0.04± 0.00)^b^	(1.15±0.02)^b^	(0.05±0.00)^a^	(0.10±0.00)^a^	
	Cv_4_	(0.6±0.4)^a^	(0.07±0.01)^a^	(1.36±0.08)^a^	(0.06±0.01)^b^	(0.21±0.00)^b^	
Cream cheese	Control	(0.8±1.3)^b^	0.11± 0.01	(0.53±0.02)^c^	0.06±0.01	(0.02±0.00)^c^	
	Cv_2_	(1.1±0.2)^b^	0.12± 0.00	(0.85±0.03)^b^	0.07±0.00	(0.10±0.00)^b^	
	Cv_4_	(2.2±0.7)^a^	0.12±0.00	(1.16±0.01)^a^	0.08±0.00	(0.16±0.00)^a^	
DRI		2.1	0.15	1.5	-	0.3	

DRI=15 % of dietary reference intake. Average values in the same column with different letters in superscript are significantly different (p<0.05)

The addition of *C. vulgaris* to the cheese increased the mass fraction of Mg, P, S, Cu, Zn, Fe and Mn. All cheese samples (control and microalga-enriched) state the nutritional claim ’source of’ calcium and phosphorus, which means that according to Regulation (EU) no 1169/2011 of the European Parliament and of the Council about the provision of food information to consumers (*38*), all the quark cheeses have 15 % of the dietary reference intake (DRI) values specified (800 and 700 mg respectively) per 100 g of the product. A significant (p<0.05) increase of phosphorus compared to control: 13.9 and 22.3 % in the quark cheese, and 7.7 and 21.1 % in the cream cheese, respectively, with the incorporation of 2 and 4 % of microalga was observed.

Calcium-binding proteins are involved in the calcium signalling pathway by binding calcium ions, which play an important role in many cellular processes (*39*). Milk αS1-, αS2- and β-caseins contain serine, glutamate, and phosphoserine residues, forming complex structures with calcium (casein micelles), improving its absorption and bioavailability in milk products (*40*). Smooth *C. vulgaris* is naturally rich in calcium (*40*) with approx. 240 mg/100 g. However, despite the high Ca mass fraction of *C. vulgaris* biomass, there was not a significant increase in the calcium mass fraction in the microalga-enriched cheese.

Regarding zinc in both quark and cream cheese and copper in the cream cheese, even though the enrichment was not enough to obtain new nutritional claims, the incorporation of microalga brought the content very close to the 15 % of the dietary reference intake value (0.15 and 1.5 mg of Cu and Zn, respectively, Table 2).

On the other hand, the cream cheese with the addition of 4 % of *C. vulgaris* can use the nutritional claim of ’source of’ iron according to Regulation (EU) no 1169/2011 (*38*). Dairy products are naturally low in iron (*41*); the increase in iron content with the incorporation of *C. vulgaris* is a major advantage, especially for consumers with vegetarian or plant-based diets.

### Total phenolic compounds and antioxidant capacity

**Fig. 1:**
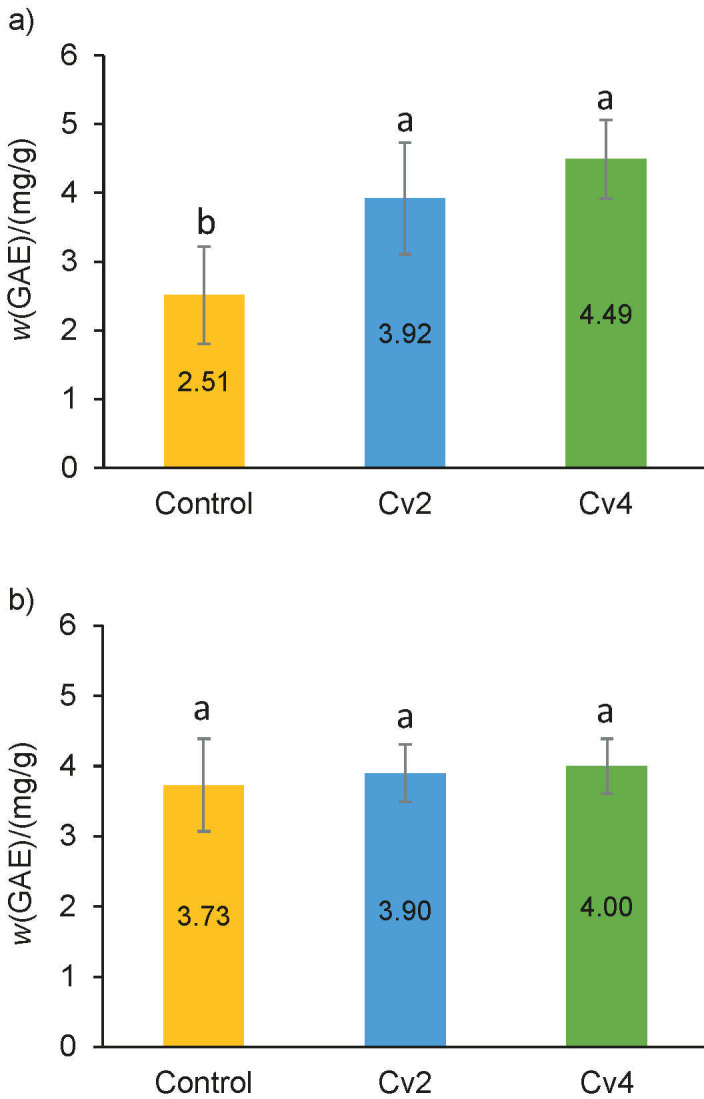
Total phenolic content on dry mass basis of: a) quark cheese and b) cream cheese enriched with 2 and 4 % of smooth *Chlorella vulgaris* (Cv_2_ and Cv_4_) compared to control cheese. Error bars indicate the standard deviations of each cheese (*N*=3). Different letters represent statistically significant differences among cheese samples

Phenolic compounds are secondary metabolites known for their antioxidant activity (*42*). The total phenolic contents (TPC) of the quark and cream cheese enriched with 2 and 4 % of *Chlorella vulgaris* are presented in Fig. 1. The TPC, expressed as GAE, of smooth *C. vulgaris* biomass (spray-dried) expressed on dry mass basis was 9.14 mg/g (data not shown). The TPC of quark cheese was higher than of the control sample for both Cv_2_ and Cv_4_ samples. Nonetheless, TPC of the cream cheese increased as a consequence of the incorporation of the microalga, but these increases were not significant (p>0.05).

**Fig. 2:**
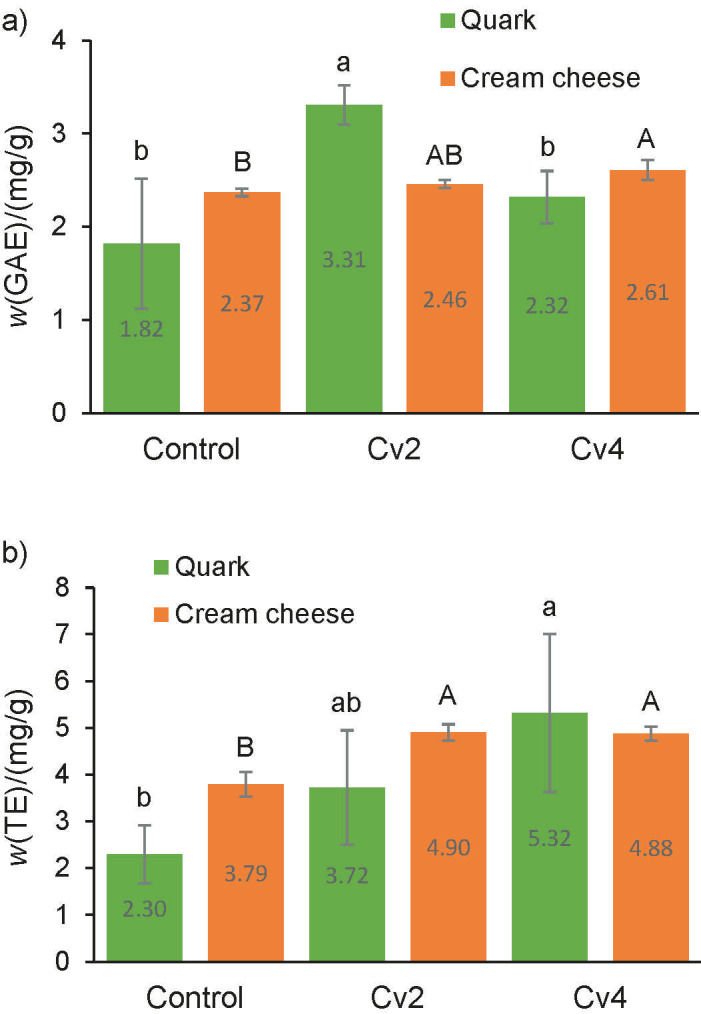
Antioxidant activity on dry mass basis of quark and cream cheese samples enriched with 2 and 4 % of smooth *Chlorella vulgaris* (Cv_2_ and Cv_4_) compared to control cheese using: a) 2,2-diphenyl-1-picrylhydrazyl (DPPH) assay expressed as gallic acid equivalents (GAE), and b) ferric reducing antioxidant power (FRAP) assay expressed as Trolox equivalents (TE). Error bars indicate the standard deviations of the cheese samples (*N*=3). Different letters represent statistically significant differences among cheese samples. Lowercase letters compare the quark cheese samples and capital letters compare the cream cheese samples

The antioxidant capacity of quark and cream cheese was evaluated using the DPPH and FRAP methods (Fig. 2[REMOVED REF FIELD]). The antioxidant activity, expressed as TE on dry mass basis, of smooth *C. vulgaris* biomass (spray-dried) was 8.17 and 35.71 mg/g for DPPH and FRAP assays, respectively (data not shown). The addition of microalga led to a significant (p<0.05) increase of the antioxidant capacity, compared to the control cheese, for both types of cheese. *C. vulgaris* has a high concentration of chlorophyll compounds such as chlorophyll a and b (*6*, *43*), lutein and its isomers (*44*). The phenolic compounds identified in *C. vulgaris* and responsible for its antioxidant activity were salicylic, *trans*-cinnamic, synaptic, chlorogenic, chimic and caffeic acids (*45*). The obtained results are in line with a study by Nunes *et al.* (*46*), where it was demonstrated that chlorophyll compounds present in the microalga *Tetraselmis chuii* have the capacity to scavenge DPPH radicals. Tohamy *et al*. (*14*) also reported a higher antioxidant activity in spreadable cheese supplemented with *C. vulgaris* than in the control.

### Texture of quark and cream cheese

**Fig. 3:**
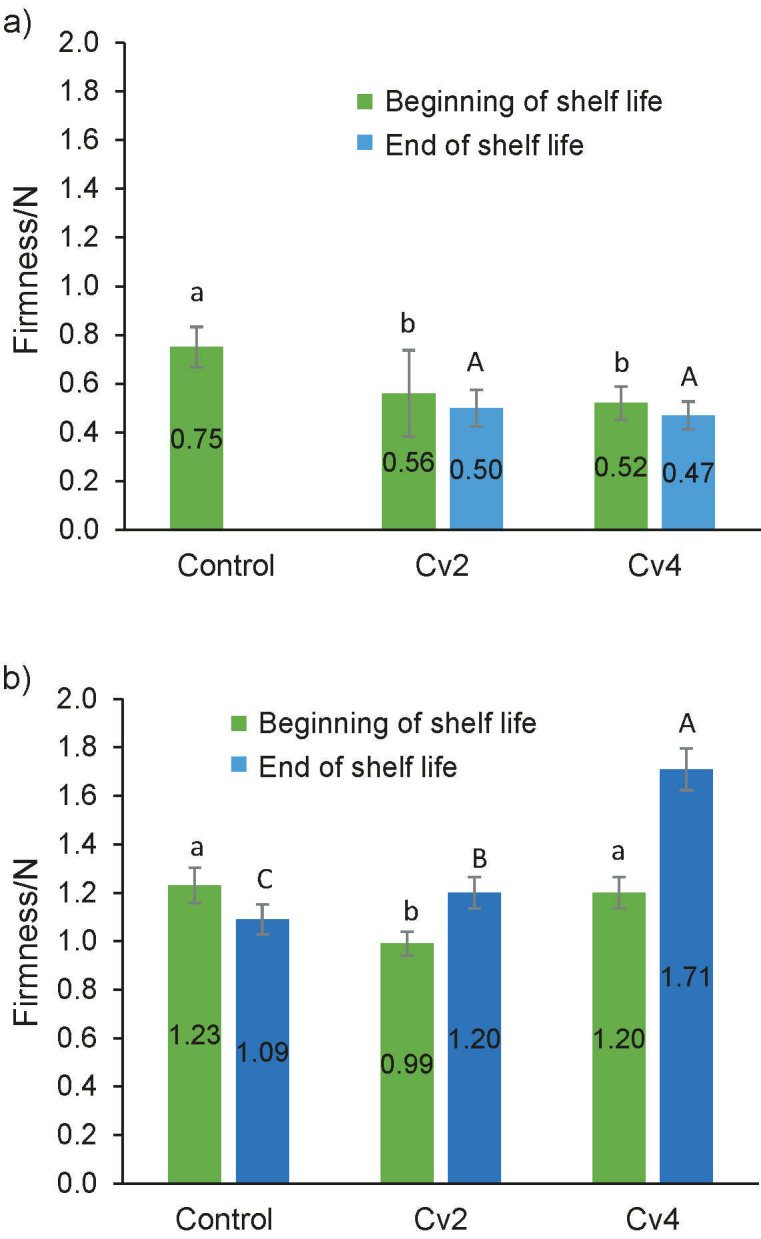
Variation of firmness of: a) quark cheese and b) cream cheese enriched with 2 and 4 % of smooth *Chlorella vulgaris* (Cv_2_ and Cv_4_) compared to control cheese. Error bars indicate the standard deviations of the cheese samples (*N*=3). Different letters represent statistically significant differences among cheese samples. Lowercase letters compare the cheese samples at the beginning of shelf life and capital letters at the end of shelf life

Fig. 3 shows the firmness of the fresh quark and cream cheese at the beginning and end of shelf life, depending on the percentage of the added smooth *C. vulgaris*.

A significant decrease (p<0.05) in firmness was observed of quark cheese with the addition of *C. vulgaris*, resulting in a softer texture than the control. However, there was no significant difference (p>0.05) between 2 and 4 % of the incorporated microalga. The decrease in firmness could be attributed to the ability of the compounds present in the microalga, particularly proteins and polysaccharides, to retain water (*47*). Consequently, the inclusion of microalgal macromolecules may enhance water retention by the casein gelled network during curd cutting and draining (*46*). The texture parameters could not be measured at the end of shelf life due to an issue with the quark control sample, resulting in missing data. However, the analysis of firmness values for quark cheese with *C. vulgaris* showed no significant differences (p>0.05) between the values obtained at the beginning and the end of the shelf-life period. This suggests that the gel of the quark cheese remained stable over the course of 45 days.

Cream cheese had higher firmness values than the quark cheese due to its composition and processing. This cheese formulation included three different stabilizers, which are hydrophilic molecules with a high molecular mass that are used to increase cheese consistency through the gelling effect, and control of the microstructure, texture, flavour and shelf life of this type of cheese (*48*). The firmness of the Cv_2_ sample decreased (1.23–0.99 N), while the Cv_4_ sample remained statistically equal to the control. At the end of shelf life, a significant decrease (p<0.05) of the control’s firmness can be observed (1.23–1.09 N in 90 days of storage), whereas there was an increase in the firmness of the cream cheese with microalga (0.99–1.20 N of Cv_2_ and 1.20–1.71 N of Cv_4_). Cream cheese is a mixture of the drained fermented curd (which results from an acid coagulation of milk) with cream, where hydrocolloids also play an important role, reinforcing the structure. This soft solid product is a dynamic gelled system, and it is prone to structural rearrangements over time. The addition of microalga to the cheese and yogurts can affect the texture parameters of these dairy products; there are authors reporting a structural reinforcement (*13*, *49*), while others observed a decrease of the gel firmness (*50*). The increase in firmness could result from the gel network formed by the caseins and strengthened by the microalgal proteins, promoting interactions between these biomolecules. Nevertheless, in some cases, there was a decrease in firmness due to the negative influence of microalgal molecules on the network formed by the casein micelles and the thickening agents. In this cream cheese, the influence of microalga at the beginning of shelf life was low, but there was a significant (p<0.05) increase in firmness with time, which can be a positive feature of *C. vulgaris* as a techno-functional ingredient.

### Rheology of quark and cream cheese

**Fig. 4:**
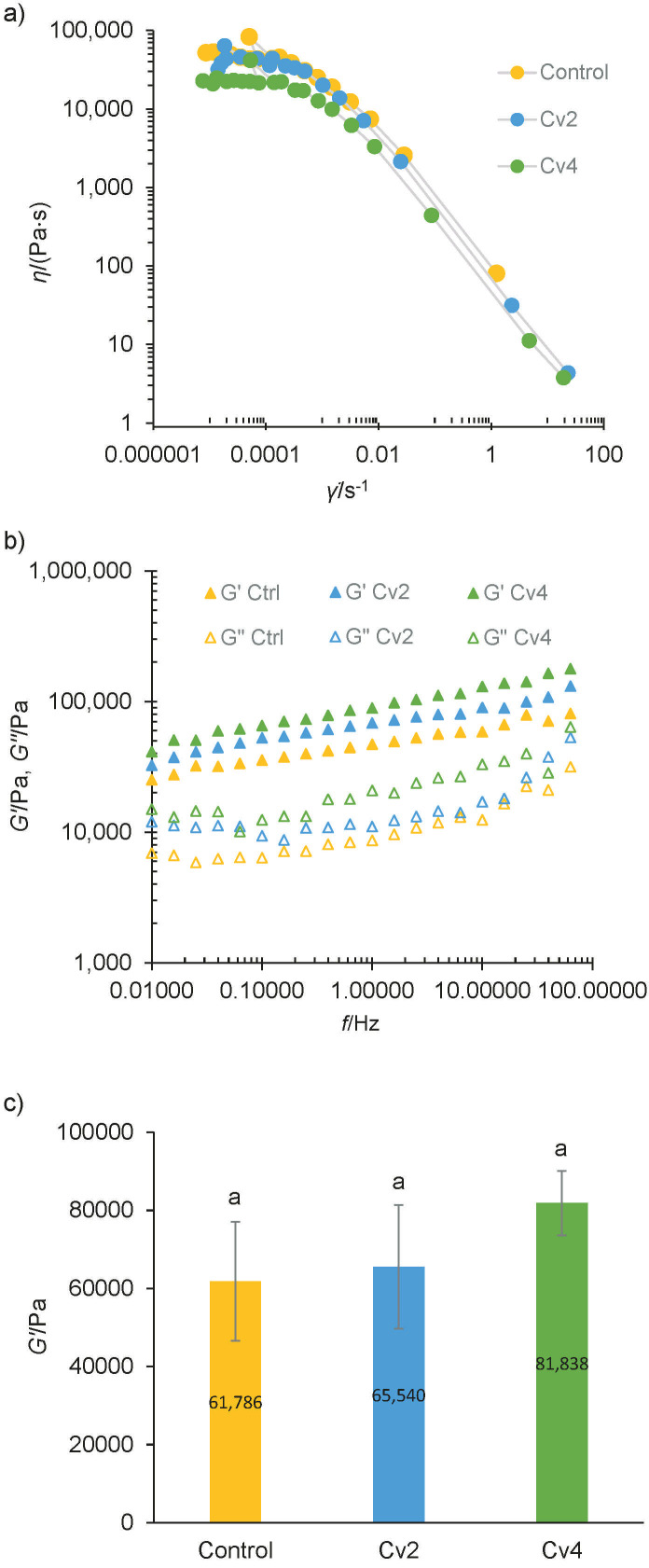
Rheological behaviour of cheese samples using: a) flow curves to analyse the quark cheese enriched with 2 and 4 % of smooth *Chlorella vulgaris* (Cv_2_ and Cv_4_) compared to control cheese, b) mechanical spectra at 5 °C of cream cheese with Cv_2_ and Cv_4_ compared to control cheese, and c) storage modulus (*G'*) at *f*=1 Hz extracted from the mechanical spectra. Error bars indicate the standard deviations of the cheese samples (*N*=3). Letters represent statistically significant differences among the cheese samples

Fresh quark cheese samples studied under steady shear conditions show similar flow curves, exhibiting a shear thinning behaviour in Fig. 4a.

Studies about the effect of microalgal biomass on the rheology behaviour of dairy products are scarce, and the results are contradictory, probably because they depend on the product composition, processing and on the characteristics of microalgae. Bchir *et al.* (*49*) reported an increase in the apparent viscosity of a yogurt enriched with 0.5 % *A. platensis*, which was attributed to the increased protein content. On the contrary, Mohammadi-Gouraji *et al.* (*51*) found that the incorporation of phycocyanin caused a decrease in the apparent viscosity, justified by the disruption of the gelled network.

In these quark cheese samples, it seems that the addition of *C. vulgaris* microalga induced a reduction in the zero-shear viscosity, which is in line with texture results; however, the cross parameters are not significantly (p>0.05) different from the control quark cheese. These results demonstrated that the addition of microalga to quark cheese does not have an impact on its flow behaviour, and therefore no changes have to be made in the manufacturing process.

A fingerprint of the viscoelastic nature of the samples can be studied by means of SAOS measurements as frequency sweeps. The mechanical spectra of the cream cheese enriched with 2 and 4 % microalga are shown in Fig. 4b. At both stages *G*’>*G*’’, which means that the storage modulus (*G'*) predominated over the loss modulus (*G*'') in the entire examined frequency range (0.01−100 Hz), indicating that all the cream cheese samples have a linear viscoelastic behaviour typical of a weak gel.

It seems that the more structured cheese was the one enriched with 4 % of smooth *C. vulgaris* as it is the cheese with higher *G*' and *G*'' than the control, although there are no differences (p>0.05) of *G*’ at *f*=1 Hz, as it can be observed in Fig. 4c.

### Colour of quark and cream cheese

The enrichment of cheese with *C. vulgaris* results in an innovative green colour due to the presence of a varied number of pigments in the microalgal biomass, mainly chlorophyll a and b and carotenoids. With the addition of smooth *C. vulgaris* to the quark and cream cheese, there was a significant (p<0.05) darkening of the samples as it can be seen in the L* reduction, and an increase in the green (a*) and yellow (b*) tonalities. Table 3 shows that the incorporation of *C. vulgaris* to both types of cheese had a significant (p<0.05) impact on all the parameters. Total colour difference (ΔE*) between Cv_2_ and Cv_4_ samples should be identified by the consumers since ΔE* value is higher than 5 (*52*).

**Table 3:** Variation of the colour parameters L*, a*, b* of fresh quark and cream cheese with 2 and 4 % of smooth *Chlorella vulgaris* (Cv_2_ and Cv_4_) compared to control cheese, and total colour difference (ΔE*) obtained at the beginning and end of shelf life

*t*(shelf life)=0							
Cheese		L*	a*	b*	ΔE*		
					Compared to control	Compared to Cv_2_	Compared to *t*=1
Quark	Control	(89.8±0.3)^a^	(-2.4±0.14)^c^	(7.8±0.5)^c^	-	-	-
	Cv_2_	(68.4±0.2)^b^	(-6.83±0.05)^a^	(32.5±0.2)^b^	32.32	-	-
	Cv_4_	(60.0±0.2)^c^	(-6.47±0.05)^b^	(36.1±0.2)^a^	40.55	9.12	-
Cream cheese	Control	(86.5±2.2)^a^	(-2.5±0.1)^c^	(14.2±0.8)^b^	-	-	-
	Cv_2_	(63.0±3.2)^b^	(-4.9±0.2)^a^	(22.0±3.8)^a^	24.88	-	-
	Cv_4_	(55.2±4.2)^c^	(-4.0±0.3)^b^	(22.0±4.8)^a^	32.26	7.83	-
*t*(shelf life)_end_=45–90 day							
		L*	a*	b*	ΔE* with control	ΔE* with Cv_2_	ΔE* with *t*0
Quark	Control	-	-	-	-	-	-
	Cv_2_	(68.0±0.5)^a^	(-6.51±0.08)^b^	(31.1±0.8)^a^	32.18	-	1.43
	Cv_4_	(60.0±0.2)^b^	(-6.07±0.05)^a^	(34.20±0.2)^b^	39.94	8.54	1.93
Cream cheese	Control	(88.2±1.8)^a^	(-2.8±0.2)^c^	(15.4±0.9)^a^	-	-	2.07
	Cv_2_	(66.8±1.8)^b^	(-5.6±0.2)^a^	(27.4±0.9)^b^	24.75	-	6.63
	Cv_4_	(58.7±0.8)^c^	(-4.9±0.4)^b^	(29.6±2.7)^b^	32.85	8.42	8.45

Mean values in the same column with different letters in superscript are significantly different (p<0.05). -=not determined

At the end of shelf life, there is a decrease of L* and a*, and a significant (p<0.05) increase of b* depending on the percentage of added microalgae.

The colour stability of each sample at the end of shelf life, compared to *t*=0 (beginning of shelf life), was observed for the quark cheese incorporated with both percentages of the microalgae (ΔE*<5) but not for the cream cheese with ΔE* higher than 5 (6.63 and 8.45 for Cv_2_ and Cv_4_, respectively). Beheshtipour *et al.* (*53*) also observed a difference in colour over time in yoghurts enriched with 0.25, 0.50 and 1.00 % of *C. vulgaris*.

### Sensory analysis of quark and cream cheese

**Fig. 5:**
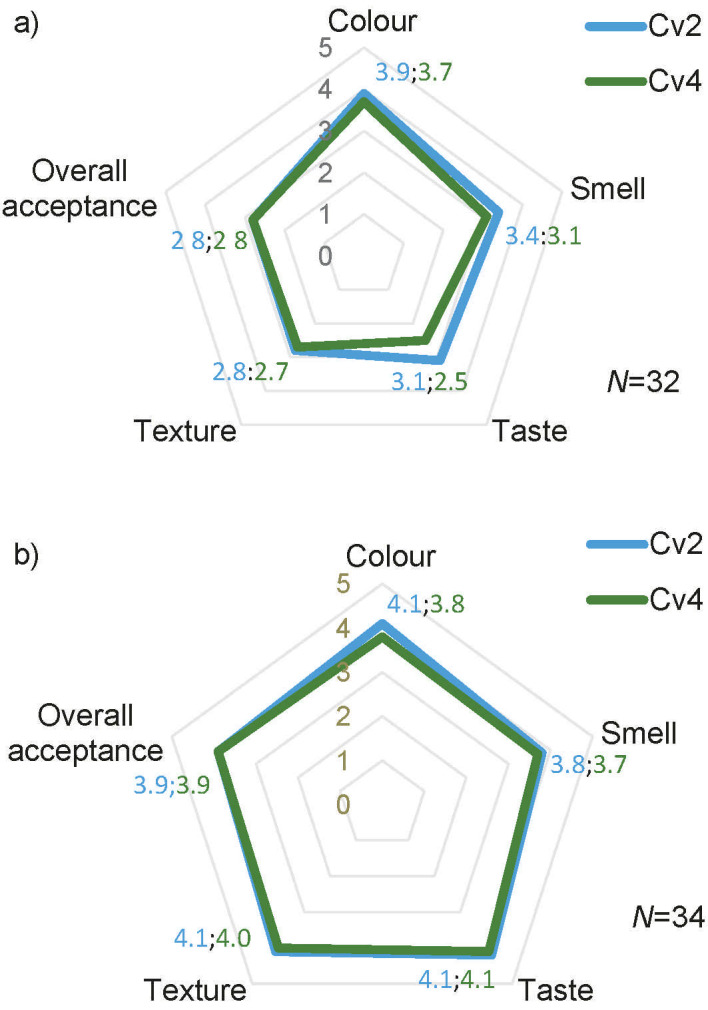
Spiderweb diagram of the sensorial analysis of: a) quark cheese and b) cream cheese incorporated with 2 and 4 % of smooth *Chlorella vulgaris* (Cv_2_ and Cv_4_)

The sensory evaluation was performed on the two types of cheese, and it showed that the quark cheese with smaller percentage of added microalga had stronger sensory acceptability. On the contrary, both percentages of microalga were similarly preferred in the cream cheese (Fig. 5).

In 2020, green coloured foods were a big trend as consumers sought products that reconnected them with nature (*54*). This colour is often associated with nutrition and health; therefore, along with the sensorial analysis, we studied the acceptance of the green colour in cheese (Table 3). The results showed that both quark and cream cheese had high scores for both *C. vulgaris* incorporations. Comparing these results with the colorimeter data in Table 3, it was found that the consumers could distinguish between the two amounts of *C. vulgaris* incorporation. This was consistent with the previous results for ΔE* (>5).

In terms of taste, the cream cheese was found to be more acceptable than quark cheese, and the difference in the acceptability between the different amounts of *C. vulgaris* was smaller among cream cheese samples. This suggests that consumers may tolerate higher amounts of *C. vulgaris* in cream cheese than in quark cheese (Fig. 5).

A previous study on the effects of the addition of *C. vulgaris* to yogurt found that increasing the amount of *C. vulgaris* from 0.25 to 1.00 % resulted in weaker sensory acceptability for all parameters (*53*).

The acceptance index for quark cheese was calculated to be 56 %, and for cream cheese it was 78 %. According to Lucas *et al.* (*55*), a product needs an acceptability index greater than 70 % to be accepted in terms of sensory characteristics. Therefore, it is expected that quark cheese containing microalgae would not be well-received by consumers, whereas cream cheese with microalgae would be well-received if commercialized.

Purchase intention was determined by using a five-point scale from 1=would certainly not buy to 5=would certainly buy. The average purchase intention of the quark cheese containing microalga was 2.7 for the Cv_2_ and 2.4 for Cv_4_, with a purchase intention index of 54 and 48 %, respectively (data not shown). For the green cream cheese, it was 3.8 and 3.6 for the Cv_2_ and Cv_4_, respectively, with a purchase intention index of 76 % for Cv_2_ and 72 % for Cv_4_. The purchase intention of the Cv_2_ cream cheese is considerably higher than the Cv_2_ quark cheese, with a higher number of panellists scoring ’would certainly buy’ and ’would probably buy’, 10 and 12 panellists respectively for cream cheese and 1 and 7 panellists, respectively, for quark cheese. The purchase intentions of both microalga-enriched cream kinds of cheese are similar and promising, although the cheese with a lower *C. vulgaris* content had a better acceptance.

## CONCLUSIONS

The protein mass fraction of quark and cream cheese with the addition of 4 % of spray-dried *Chlorella vulgaris* (Cv) biomass increased by 21.2 and 6.9 % respectively, compared to the control. There was an important increase in Zn and Cu mass fractions, and the cream cheese with 4 % Cv can state the nutritional claim ’source of Fe’. Although there are some changes in the firmness, sensory results are promising, especially with a purchase intention index of 76 and 72 %, respectively, of the cream cheese samples with 2 and 4 % of microalgal incorporation. As this work is related to the improvement of the cheese nutritional profile, it suggests that the green microalga *C. vulgaris* can be used as a promising functional ingredient of vegetable origin for the enrichment of traditional dairy products, obtaining innovative and more sustainable products, with a greater antioxidant capacity, protein content and mineral profile, and good sensory acceptance.
